# Contraceptive use and method mix dynamics in Sub-saharan Africa: time trends and the influence of the HIV pandemic

**DOI:** 10.1186/s40834-024-00273-z

**Published:** 2024-04-09

**Authors:** Ayaga A. Bawah, Pearl S. Kyei, Charles Agyei-Asabere

**Affiliations:** Regional Institute for Population Studies, University of Ghana, Post Office Box LG 96, Legon, Accra, Ghana

**Keywords:** Contraceptive method mix, Contraceptive choice, Barrier methods

## Abstract

**Background:**

Contraceptive use dynamics continue to be of priority in sub-Saharan Africa because of persistently high levels of fertility. This paper focuses on the use of barrier versus non-barrier contraceptive use in sub-Saharan Africa hypothesizing that the HIV pandemic in the region would be responsible for increases in the use of barrier methods over time.

**Methods:**

This paper uses Demographic and Heath Survey (DHS) data from 32 countries to conduct extensive analysis of trends in contraceptive use and method mix that refers to the distribution of contraceptive methods use among the sexually active population. The paper examines how contraceptive method mix dynamics have changed over time and whether the trends differ by marital status and gender using cross-tabulations. It furthers examines the determinants of method choice using logistic regressions.

**Results:**

The findings indicate that the use of barrier methods, most markedly for unmarried women and men, rose substantially between the late 1980s and late 2000s in the region in tandem with trends in HIV prevalence. The results further show marked differences in method mix by gender with men being more likely to report barrier method use than women.

**Conclusions:**

The findings indicate shifting preferences in contraceptive choice. The time trend analyses highlight the importance of expanding the focus of contraceptive use studies beyond women in this context as the study finds differing trends for men.

**Graphical Abstract:**

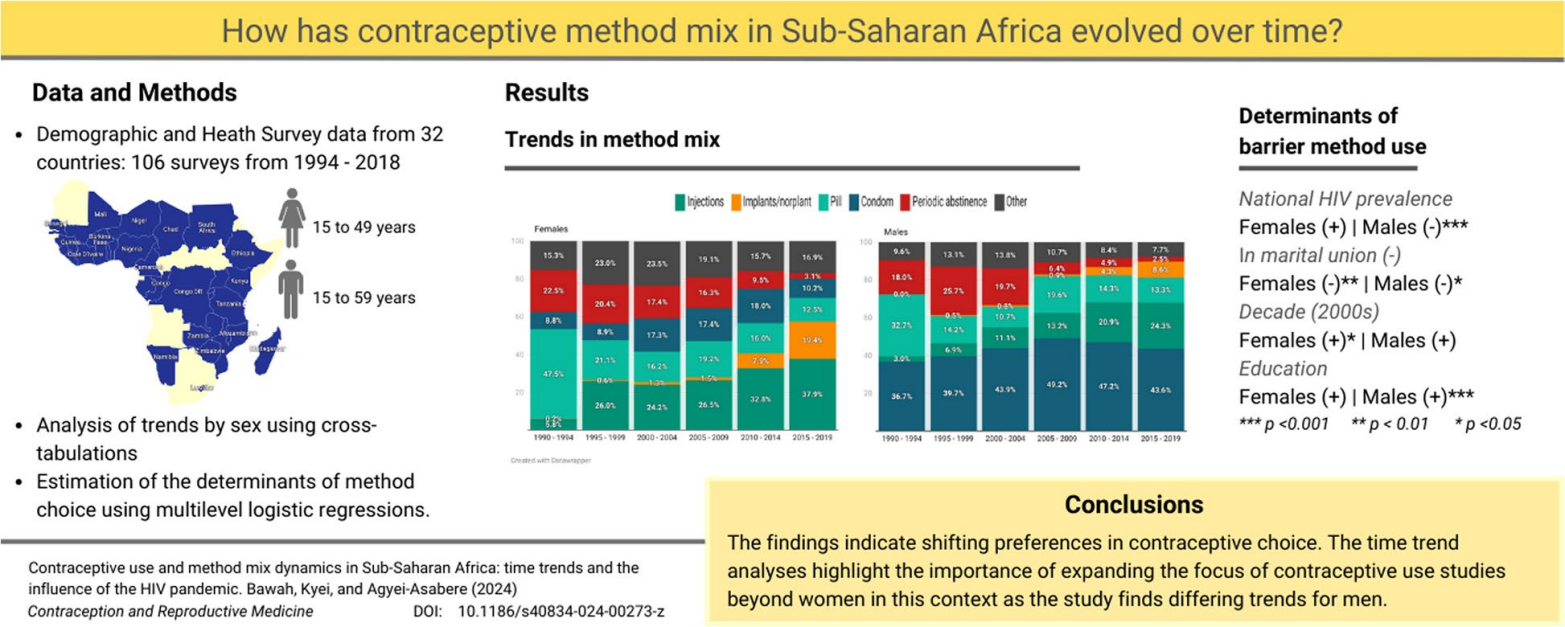

## Background

This paper analyses trends in contraceptive use and method mix dynamics in sub-Saharan Africa. Method mix refers to the distribution of contraceptive use among the sexually active population i.e. the percentage share of each contraceptive method that is being used. The study of contraceptive use dynamics continues to be of priority in sub-Saharan Africa because of the high level of fertility in many countries of the region despite various interventions to reduce fertility. Following results from the World Fertility Surveys and subsequently, the Demographic Health Surveys, showing high levels of fertility in sub-Saharan Africa, family planning programs became widespread throughout the region with the aim of reducing fertility.

As a result, rapid fertility reductions were initially recorded in many countries [1, 2] though subsequent decades have seen the uptake of contraceptive use slowing down in most countries [3, 4]. This slowdown accompanied by stalling fertility has raised concerns over declining interest in family planning. More specifically, the area of contraceptive method mix has become of specific importance because it provides perspectives on both the demand and supply side determinants of contraceptive prevalence. For instance, a method mix skewed towards one method can be indicative of limited access to other options or cultural preferences drawing women towards that method at the exclusion of others. Understanding the factors that drive contraceptive choices represents an integral part of increasing contraceptive prevalence in this context.

Some contraceptive methods i.e. male and female condoms, in addition to be being methods for planning, are also methods for preventing sexually transmitted infections (STIs) and protecting the reproductive health of sexually active persons. This paper studies contraceptive use dynamics in sub-Saharan Africa over the past three decades with a focus on barrier methods as those that prevent STIs in addition to unwanted pregnancies i.e. male and female condoms. The analysis first examines the overall trends in method mix then narrows in on the determinants of condom usage over time.

The study thus seeks to answer the following research questions: First, how have contraceptive method mix dynamics changed over time? We hypothesize that the HIV pandemic in the region would drive an increase in the use of barrier methods over the period as sexually active adults increasingly seek protection against STIs/HIV. The second research question is whether perceived HIV risk is a determinant of method choice? We hypothesize that the choice of a barrier versus non-barrier method will be influenced by perceptions of HIV risk. Sexually active adults would be more likely to choose condoms over other methods where they perceive the risk of contracting HIV is high i.e., they live in high prevalence country and/or they know that risky sex increases the likelihood of transmission. Some literature has documented the positive relationship between perception of HIV risk and condom usage in this setting [5–8].

The third research question is whether these trends differ for married versus unmarried and for men versus women? We hypothesize that married women would be less likely to use barrier methods as they are presumably in monogamous relationships and men would be more likely to use barrier methods as they are more likely to have multiple sexual partners and perceive themselves as lower risk [5, 6]. The literature predominantly focuses on the contraceptive behaviour of married women and as such, we aim to make an additional contribution by studying the trends for unmarried women and men as well.

The findings from this multi-country study will contribute to the existing literature on contraceptive use in sub-Saharan Africa by providing an overview of how contraceptive method mix has evolved over time in this context. In addition, it will provide additional insight on changes over time in the demand side influences of contraceptive choice, with a particular focus on the influence of the HIV pandemic.

Research on contraceptive trends in developing countries has shown changes in the contraceptive method mix in these regions [1, 9–11] – an increasing use of barrier methods (including condoms) with other methods (such as the oral pill, and intrauterine devices) becoming less popular. Other non-barrier methods such injectables have increased in popularity as well. The increasing use of barrier methods suggests that contraceptive use is increasingly more related to protection against sexually transmitted infections (STIs) generally and HIV/AIDS in particular. Despite ample evidence for the changing trends, the literature has not adequately studied the reasons for the shifts.

This paper focuses on the relative mix of barrier methods that protect against STIs versus other methods given the hypothesis that the HIV pandemic would increase usage of the former and the subsequent introduction of ARTs would decrease perceived risks of HIV and thus the usage of barrier methods. Method mix is determined by both demand and supply side factors – individuals choose methods based on personal preferences and budget constraints while the availability of methods will determine how much they are used. Despite the ample literature on contraceptive use patterns and trends in sub-Saharan Africa, there is relatively little on the factors explaining the changes in contraceptive method mix. This paper focuses on one demand side factor which is the preference for barrier methods based on the assumption that HIV perceptive risk will increase the demand for those methods.

## Methods

Country-level data on HIV prevalence during the survey year was obtained from the Joint United Nations Programme on HIV and AIDS (UNAIDS) database which provides annual HIV prevalence estimates from the year 1993.

Individual-level data was from the Demographic and Health Survey (DHS) [12]: the female [IR] and the male [MR] datasets. sample comprised women aged 15 to 49 years and males 15 to 59 years. The data was pooled from 106 surveys. To study time trends, the sample was restricted to 32 sub-Saharan African countries that had conducted a minimum of two standard DHS from 1994 when the HIV prevalence estimates were available. The surveys were: Benin(5), Burkina Faso(3), Burundi(2), Cameroon(3), Chad(3), Comoros(2), Congo(2), Congo DR(2), Cote D’Ivoire(3), Ethiopia(4), Gabon(2), Ghana (4), Guinea(4), Kenya(4), Lesotho(3), Liberia(2), Madagascar(3), Malawi(4), Mali(5), Mozambique(3), Namibia(3), Niger(3), Nigeria(4), Rwanda(4), Senegal(4), Sierra Leone(2), South Africa(2), Tanzania(4), Togo(2), Uganda(5), Zambia(5), Zimbabwe(5). The sample comprised respondents who had been sexually active in the last 12 months preceding the survey^1^. Due to non-proportional sampling from various regions in urban and rural areas, sample weights were applied to make surveys nationally representative.

The dichotomous outcome variable for this study is usage of a barrier contraceptive method with most recent sexual partner. Barrier method in this study is defined as a method that is physical barrier against STI transmission i.e., condom use (male or female). This definition differs from the more conventional definition of barrier method which refers to contraception that prevents sperm from entering the uterus because of the hypothesis that increasing importance of HIV prevention is a key driver to method mix trends over time.

To study the time trends in the method mix, we run country-level frequencies of contraceptive use over time by five-year intervals. We first present the dynamics for all methods before presenting trends in barrier usage compared to non-barrier. Surveys are usually conducted every five years; however, some countries had their next DHS conducted outside the five-year range. To show a trend in the absence of data would distort findings, and as such Warring Lagrange interpolation method was used to find an estimate of an indicator by using two data points. The limitation of this mode of interpolation method is the risk of not yielding good estimates where there are relatively few data points and the intervals between the points is wide as is the case for a limited number of countries in the sample.

Surveys conducted before 1995 and Tanzania 2016 were excluded from the analysis because the DHS in those years did not ask questions on knowledge of HIV, a key explanatory variable for this analysis. Male surveys for Cameroon, the Democratic Republic of Congo, Gabon, Mali, Niger, and South Africa were excluded from the analysis because questions on contraceptive used were not asked or there was no variation in the responses when asked i.e., 100% of users were using either modern or traditional methods.

We applied a multilevel logistic regression model to explore individual, contextual (level of HIV, period of survey) and country level factors associated with barrier method (condom) usage. A logistic regression is used because the outcome variable for the analysis is a binary one and the multilevel approach is used because the pooled data has a hierarchical structure with women nested within countries in the sample.

The analysis of the pooled sample is based on a two-level logit model that accounts for clustering in the data, with level 1 defined by individuals, and level 2 by country in which the surveys were conducted. The study used a logit model based on the binary nature of the outcome variable under study (barrier method usage Y = 1 if participant used condom and 0 if otherwise).

‘Null’ binary logistic regression model – no independent variables included:

$${logit \,Pr(usage \,of \,barrier \,method }_{ijk}=yes )={\beta }_{0}+{\beta }_{\begin{array}{c}0i \\ \end{array}}+{\beta }_{0ij}$$

‘Intermediate’ binary logistic regression model – with selected Country, HIV level and Survey period level independent variables:

$${logit\;Pr(usage\;of\;barrier\;method}_{ijk}=yes\left|selected\;variables\right)=\beta_0+{\beta_{\begin{array}{c}0i\\\end{array}}+\beta_{0ij}+\beta}_1{\text{C}\text{o}\text{u}\text{n}\text{t}\text{r}\text{y}}_i{+\beta}_2{\text{H}\text{I}\text{V l}\text{e}\text{v}\text{e}\text{l}}_{ij}{+\beta}_3{\text{S}\text{u}\text{r}\text{v}\text{e}\text{y p}\text{e}\text{r}\text{i}\text{o}\text{d}}_{ij}$$

‘Full’ binary logistic regression model – with selected Country, HIV level and Survey period and individual-level independent variables:

$${logit\;Pr(usage\;of\;barrier\;method}_{ijk}=yes\left|selected\;variables\right)=\beta_0+{\beta_{\begin{array}{c}0i\\\end{array}}+\beta_{0ij}+\beta}_1{\text{C}\text{o}\text{u}\text{n}\text{t}\text{r}\text{y}}_i{+\beta}_2{\text{H}\text{I}\text{V l}\text{e}\text{v}\text{e}\text{l}}_{ij}{+\beta}_3{\text{S}\text{u}\text{r}\text{v}\text{e}\text{y p}\text{e}\text{r}\text{i}\text{o}\text{d}}_{ij}{+\beta}_4{\text{A}\text{i}\text{d}\text{s k}\text{n}\text{o}\text{w}\text{l}\text{e}\text{d}\text{g}\text{e}}_{ij}+\beta_5{\text{M}\text{a}\text{r}\text{i}\text{t}\text{a}\text{l s}\text{t}\text{a}\text{t}\text{u}\text{s}}_{ij}+\beta_6\text{E}\text{d}\text{u}\text{c}\text{a}\text{t}\text{i}\text{o}\text{n}\text{a}\text{l l}\text{e}\text{v}\text{e}\text{l}{}_{ij}+\beta_7{\text{A}\text{g}\text{e g}\text{r}\text{o}\text{u}\text{p}}_{ij}+\beta_8{\text{W}\text{e}\text{a}\text{l}\text{t}\text{h s}\text{t}\text{a}\text{t}\text{u}\text{s}}_{ij}+\beta_9{\text{R}\text{e}\text{l}\text{i}\text{g}\text{i}\text{o}\text{n}}_{ij}+\beta_{10}{\text{T}\text{y}\text{p}\text{e o}\text{f p}\text{l}\text{a}\text{c}\text{e o}\text{f r}\text{e}\text{s}\text{i}\text{d}\text{e}\text{n}\text{c}\text{e}}_{ij}+\beta_{11}{\text{N}\text{u}\text{m}\text{b}\text{e}\text{r o}\text{f l}\text{i}\text{v}\text{i}\text{n}\text{g c}\text{h}\text{i}\text{l}\text{d}\text{r}\text{e}\text{n}}_{ij}$$

The analysis required pooled data (multiple surveys from different sub-Saharan African countries), the standard DHS weights v005 were denormalized as follows:

$${weight\;Denormalized}^\ast=v005\;\text{X}\frac{\left(total\;female\;population\,15-49\;at\;period\;of\;survey\right)}{\left(total\;number\;of\;females\;interviewed\;insurvey\right)}$$

Weights applied conformed to the DHS specification for a pooled data.^2^

The key explanatory variables for the multivariate analyses are HIV prevalence in country of residence and respondent knowledge of HIV/AIDS and education. HIV prevalence in country of residence is a continuous variable that represents the UNAIDS prevalence estimate in the year the DHS was conducted. Countries were grouped into two categories: ‘low prevalence’ for countries that had a prevalence below 5% and ‘high prevalence’ for those with prevalence of 5% or above.

HIV knowledge is represented by a variable with four categories. The DHS asks respondents a variety of questions to assess their knowledge of HIV/AIDS such “ever heard of AIDS”, or “can someone get HIV by sharing food”. Questions were recoded so that all correct answers were scored 1. The percentage score on the HIV knowledge questions were grouped into three categories: Less than 50% was “low knowledge”, score of 50 to 79.9% was classified as “fair knowledge” and score of 80–100% was classified as “strong knowledge”.

The regression models also control for respondent age, marital status, religion, type of place of residence, and country as well as the year of the survey. Marital status was grouped into three categories: never married for respondents who had never been in any union before, in union for currently married or living in union and formerly married for those who were separated, divorced, or widowed. Number of surviving children was grouped into none, 1–3 children, 4–6 children and 7 or more. Survey year was grouped into five-year intervals starting from 1990 to 1994 and ending with 2015 to 2019. The standard DHS coding were maintained for these variables, age group, education, wealth status and type of place of residence (Table 1).

**Table 1:** Sample descriptive characteristics

Indicator variables	Male			Female		
	Barrier	Non-barrier	Total	Barrier	Non-barrier	Total
**HIV Prevalence in Survey Year**						
Low	56.97	43.03	41,556	23.42	76.58	89,956
High	50.31	49.69	58,970	15.36	84.64	124,306
**HIV/AIDS Knowledge**						
Low knowledge	59.69	40.31	19,646	17.74	82.26	53,535
Fair knowledge	54.23	45.77	37,930	19.21	80.79	78,884
Strong knowledge	48.44	51.56	45,799	18.32	81.68	84,938
**Survey Period**						
1990–1994	49.24	50.76	1,168	13.20	86.80	2,364
1995–1999	60.47	39.53	7,150	13.29	86.71	20,428
2000–2004	60.95	39.05	8,462	24.01	75.99	23,698
2005–2009	56.56	43.44	17,289	23.99	76.01	35,251
2010–2014	52.56	47.44	40,204	21.76	78.24	81,332
2015–2019	46.44	53.56	29,106	11.13	88.87	59,169
**Marital Status**						
Never married	89.60	10.40	39,287	50.95	49.05	40,277
Married or living together	26.62	73.38	59,288	10.47	89.53	163,209
Formerly married	72.88	27.12	4,802	20.50	79.50	18,750
**Educational Status**						
No education	42.04	57.96	9,909	6.55	93.45	37,459
Primary	45.55	54.45	34,961	11.90	88.10	85,697
Secondary	58.99	41.01	45,784	27.84	72.16	83,972
Higher	58.03	41.97	12,720	35.91	64.09	15,112
**Age Category**						
15–19	91.49	8.51	10,100	45.24	54.76	20,083
20–24	79.37	20.63	20,508	26.14	73.86	45,589
25–29	56.96	43.04	20,399	16.45	83.55	49,505
30–34	40.68	59.32	16,553	12.62	87.38	40,795
35–39	32.64	67.36	13,230	11.28	88.72	33,042
40–44	28.37	71.63	10,225	10.41	89.59	21,711
45–49	27.16	72.84	7,107	10.22	89.78	11,517
50–54	27.34	72.66	4,026	-	-	-
55–59	29.52	70.48	1,232	-	-	-
**Wealth Status**						
Poorest	44.95	55.05	10,617	10.97	89.03	25,379
Poorer	46.13	53.87	14,854	13.78	86.22	33,166
Middle	50.15	49.85	18,147	15.90	84.10	40,166
Richer	52.96	47.04	24,888	19.28	80.72	52,603
Richest	59.01	40.99	34,874	24.78	75.22	70,928
**Religious Affiliation**						
No religion	43.23	56.77	4,799	16.29	83.71	3,924
Christian	51.28	48.72	77,903	19.93	80.07	175,248
Muslim	62.30	37.70	17,281	12.34	87.66	37,899
Traditionalist/Animist	45.87	54.13	2,022	16.86	83.14	2,200
Other	56.55	43.45	1,306	28.62	71.38	2,813
**Place of Residence**						
Urban	60.48	39.52	46,520	26.16	73.84	100,817
Rural	46.34	53.66	56,859	12.43	87.57	121,425
**Number of Living Children**						
None	88.31	11.69	38,859	65.33	34.67	27,777
1–3 children	36.53	63.47	38,244	15.00	85.00	119,238
4–6 children	24.48	75.52	18,797	7.78	92.22	60,988
7 or more	21.29	78.71	7,480	4.83	95.17	14,239
**Country **						
Benin	81.04	18.96	2,318	30.25	69.75	5,100
Burkina Faso	74.27	25.73	2,917	30.21	69.79	4,036
Burundi	39.44	60.56	1,579	7.81	92.19	3,546
Cameroon	-	-	-	61.24	38.76	6,651
Chad	67.01	32.99	727	16.13	83.87	1,039
Comoros	79.04	20.96	564	19.81	80.19	762
Congo	-	-	-	71.97	28.03	3,364
Congo DR	-	-	-	61.87	38.13	2,193
Cote D’Ivoire	84.30	15.70	1,849	37.16	62.84	2,157
Ethiopia	17.91	82.09	7,180	1.83	98.17	9,057
Gabon	-	-	-	70.39	29.61	2,969
Ghana	58.56	41.44	3,098	22.01	77.99	3,755
Guinea	86.79	13.21	1,429	24.05	75.95	2,752
Kenya	43.49	56.51	9,185	8.05	91.95	18,242
Lesotho	78.30	21.70	2,937	32.29	67.71	7,828
Liberia	60.28	39.72	2,985	9.67	90.33	4,755
Madagascar	22.24	77.76	1,777	5.61	94.39	5,629
Malawi	46.44	53.56	7,717	7.16	92.84	24,051
Mali	-	-	-	5.54	94.46	4,899
Mozambique	66.11	33.89	1,023	19.09	80.91	4,814
Namibia	74.26	25.74	5,523	35.89	64.11	11,483
Niger	-	-	-	1.73	98.27	1,980
Nigeria	83.63	16.37	7,154	36.37	63.63	12,913
Rwanda	28.37	71.63	3,940	8.47	91.53	8,185
Senegal	73.93	26.07	2,732	9.98	90.02	4,570
Sierra Leone	37.71	62.29	3,327	3.12	96.88	7,793
South Africa	-	-	-	12.68	87.32	9,740
Tanzania	58.87	41.13	2,380	17.20	82.80	5,790
Togo	78.34	21.66	1,722	36.53	63.47	2,261
Uganda	55.64	44.36	3,573	15.38	84.62	9,876
Zambia	50.70	49.30	12,961	13.92	86.08	14,087
Zimbabwe	34.81	65.19	12,784	7.77	92.23	15,964

## Results

### Time trends in contraceptive method mix (Figure 1a and b)

The figures present method mix dynamics over time by gender. For both males and females, the method mix has changed substantially over time with reported usage of the pill, which had the highest proportion of usage at the start of the period, declining over time. Use of IUDs also declined over time while there was increasing usage of implants as a longer-term contraception in place of sterilization which declined over time. We see the usage of condoms increase in the mid-1990s to mid-2000s then decline after.

**Fig. 1:**
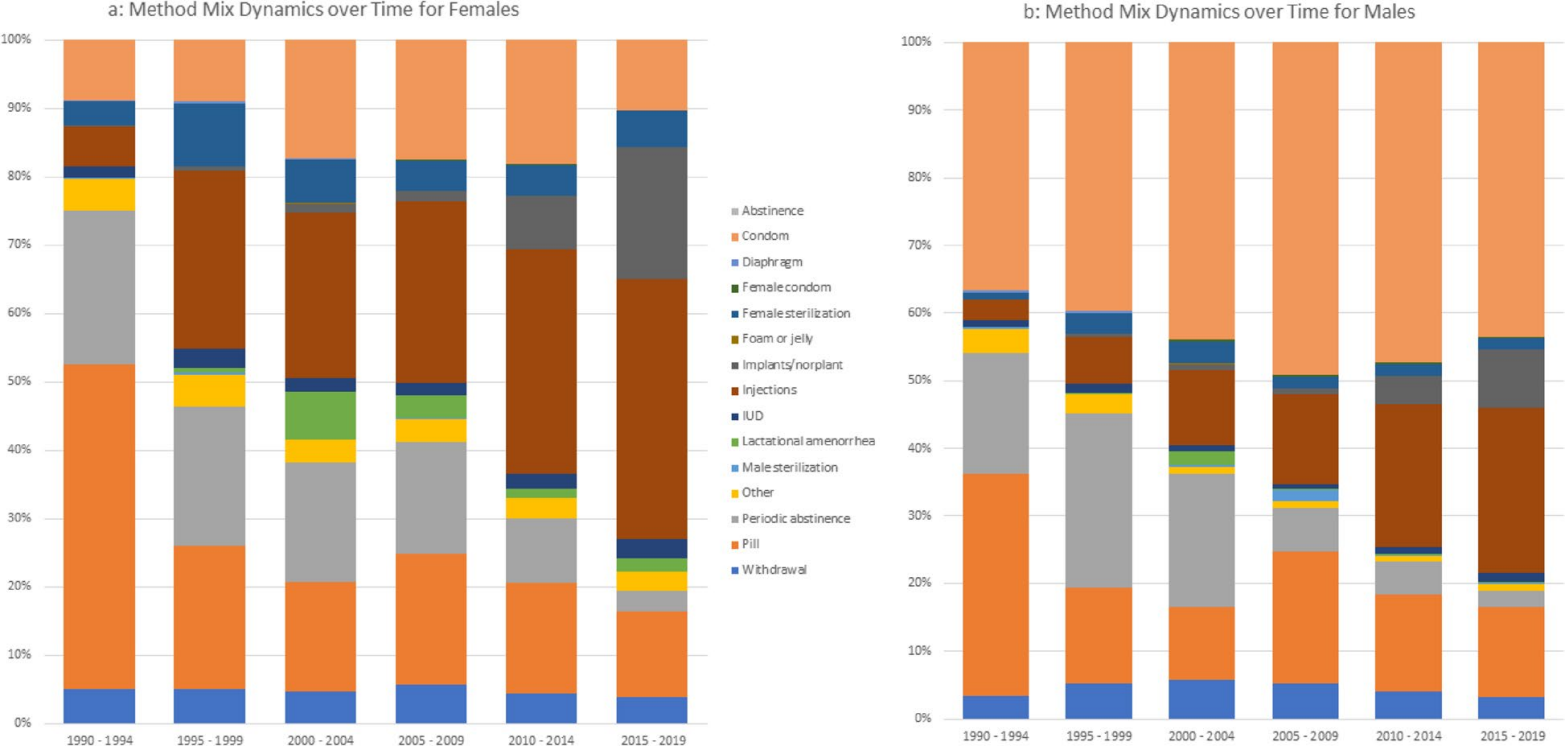
**a** Method mix dynamics over time for females. **b** Method mix dynamics over time for males

The increased usage of condoms in mid-1990s at the height of the HIV/AIDS pandemic is likely due to increase in demand for contraceptives to protect from STI infection as opposed to unwanted pregnancies. The decline from the mid-2000s coinciding with the rollout of antiretroviral therapy programmes could be attributed to changing perceptions on the deadliness of the disease and by extension decreasing commitment to HIV prevention behaviour. The decline in abstinence could be attributed to both demand (changing preferences and increased knowledge) and supply side (increased access to modern contraception) factors. The reduction in the use of the pill alongside the increase in the late 1990s injections and the implant in the 2010s can be indicative of increased preference for longer-term acting contraceptives.

**Fig. 2:**
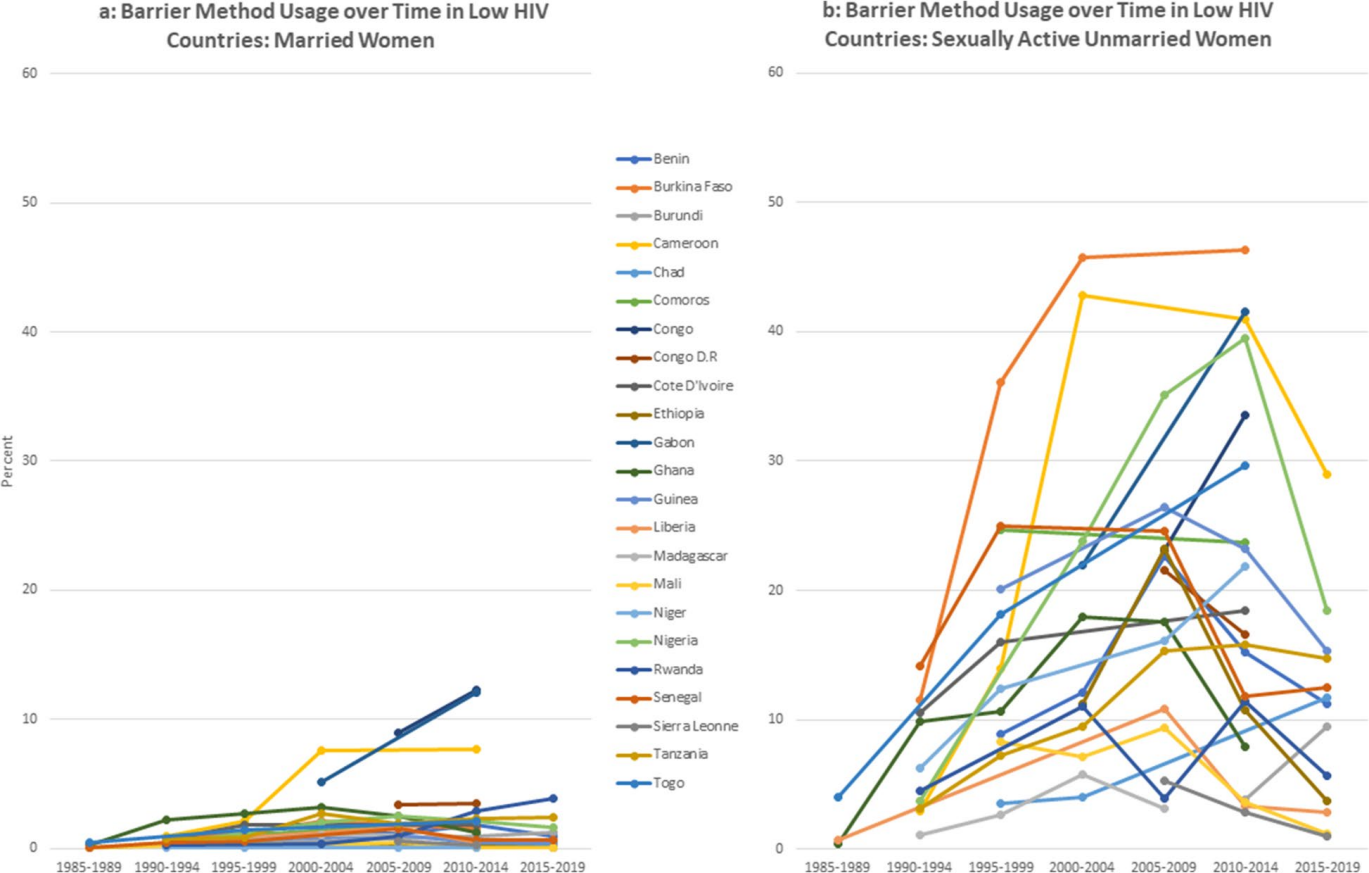
**a** Barrier method usage over time in low HIV countries: married women. **b** Barrier method usage over time in low HIV countries: sexually active unmarried women

**Fig. 3:**
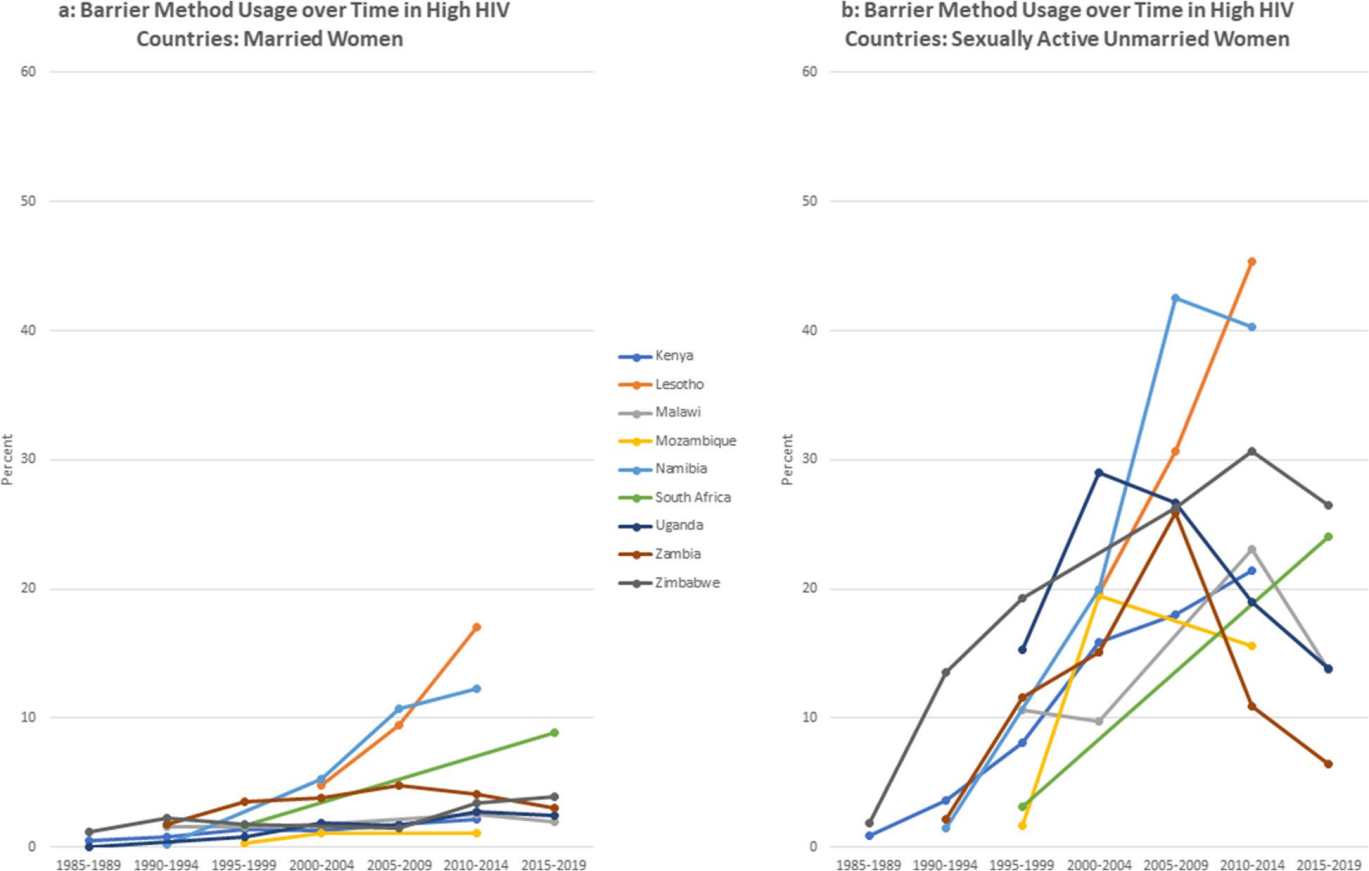
**a** Barrier method usage over time in high HIV countries: married women. **b** Barrier method usage over time in high HIV countries: sexually active unmarried women

**Fig. 4:**
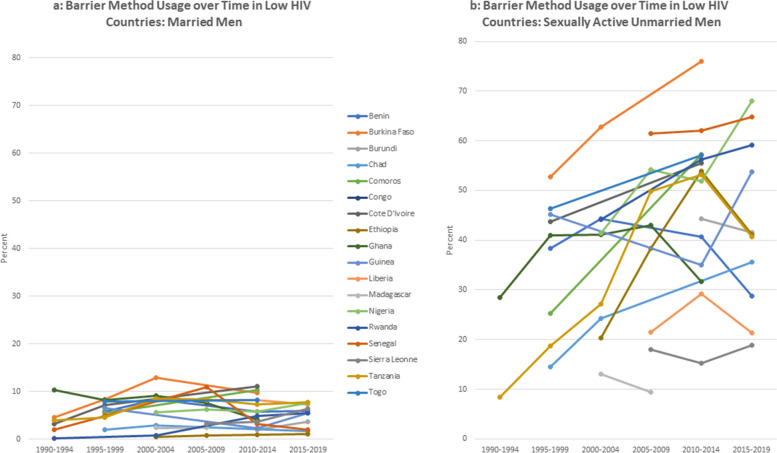
**a** Barrier method usage over time in low HIV countries: married men. **b** Barrier method usage over time in low HIV countries: sexually active unmarried men

**Fig. 5:**
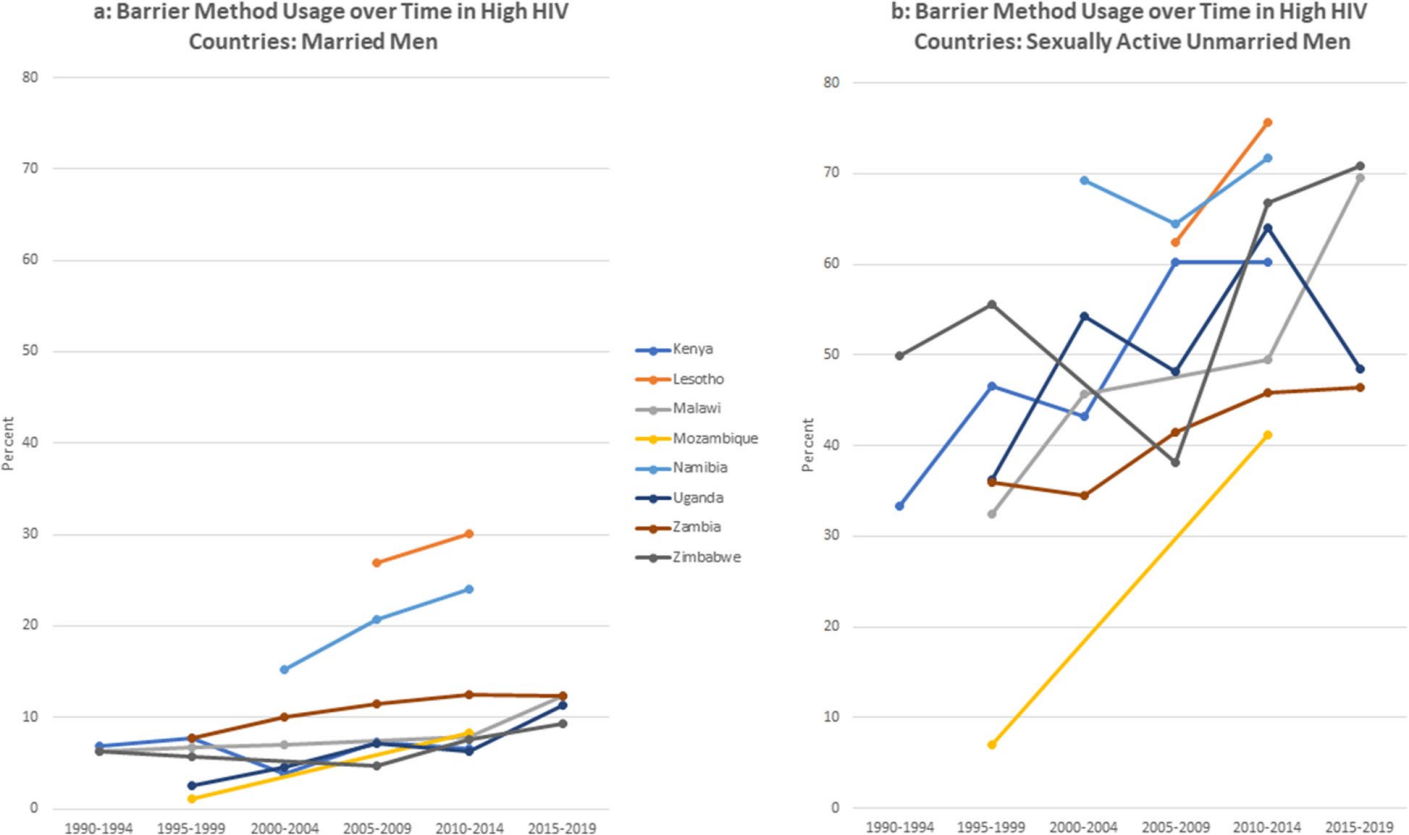
**a** Barrier method usage over time in high HIV countries: married men. **b** Barrier method usage over time in high HIV countries: sexually active unmarried men

There are gender differences in the dynamics over time – most noticeably in the use of injections and condoms. Injections for women increased over the time and by the most recent period, was the most used option among women was the injection. Men reported significantly higher usage of condom use than women. For men, condoms were consistently the most used contraceptive choice (Figs 2a, 3, 4 and 5b).

The figures compare trends in barrier method use over time for married and unmarried and women. For this analysis, countries are classified using mean HIV prevalence over the period into high prevalence (5% and above) and low prevalence (less than 5%).

As expected, the barrier method usage among unmarried women is generally higher than those in union, both in low and high HIV settings. Noticeably, barrier method usage among the unmarried women rose substantially up to the middle of the period and thereafter began to decline. Usage rates among women in union are, however, lower whether in low or high HIV setting, and relatively more stable over the period compared to the unmarried sexually active women. This is to be expected as more married women would be less likely to be using condoms with their partners compared to those who are unmarried. On the other hand, sexually active women who are not in any union are likely to use barrier methods, either to prevent pregnancy or sexually transmitted infections.

For men, we observe a similar pattern whereby barrier method usage is low among married men both in low and high HIV settings, compared to those that are not married. In Namibia and Lesotho, sexually active men have slightly higher use rates in the high HIV categories relative to the others in that category.

### Couple concordance in reported contraceptive method

The differences in the method mix of males versus females are striking. We hypothesize two possible explanations for the observed gender differences in contraceptive method mix –reporting of clandestine contraceptive use by women wanting to limit their births without their partner’s knowledge and men having multiple concurrent partners and using condoms with some partners. To further understand this phenomenon, we use data from the DHS couples file which presents couple-level data on contraceptive usage.

**Fig. 6:**
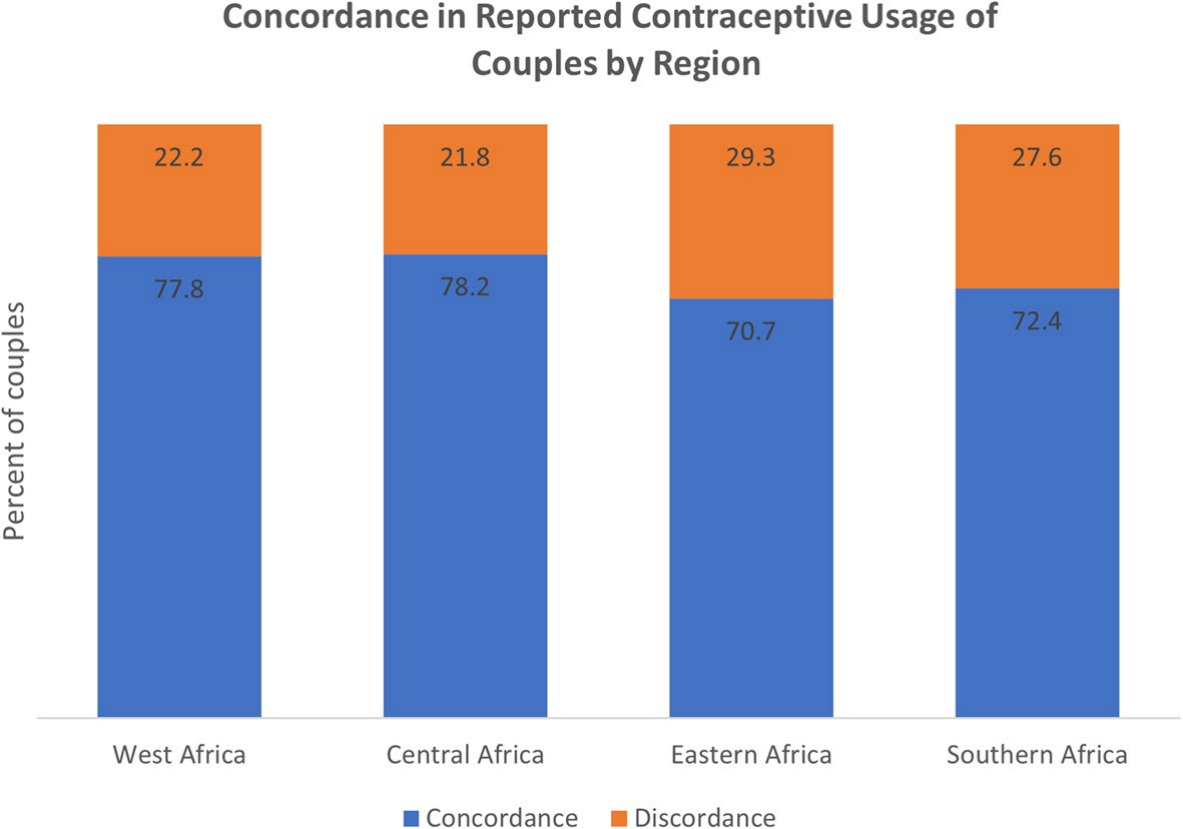
Concordance in reported contraceptive usage of couples by region

**Fig. 7:**
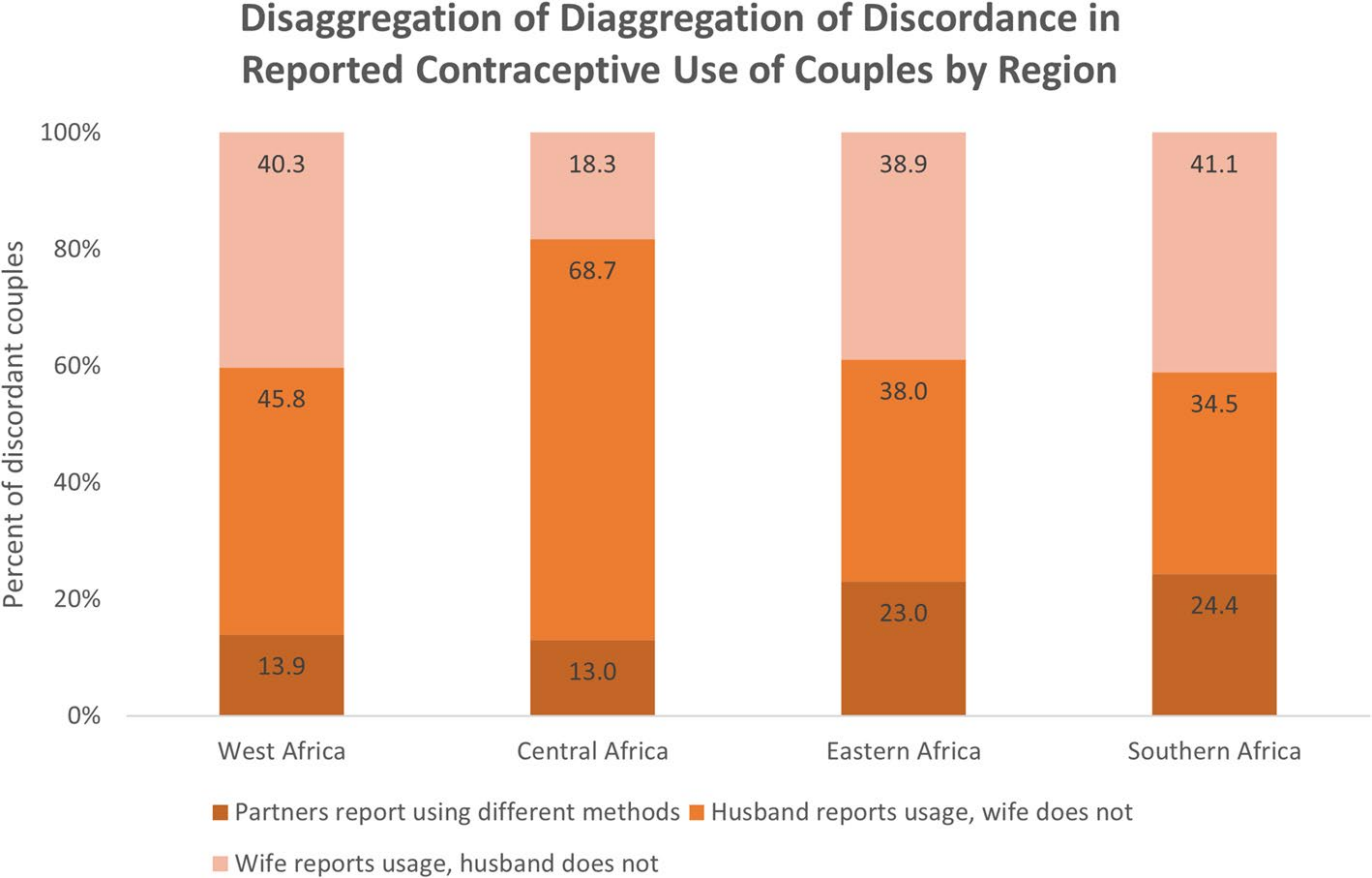
Disaggregation of diaggregation of discordance in reported reported contraceptive usage of couples by region

**Fig. 8:**
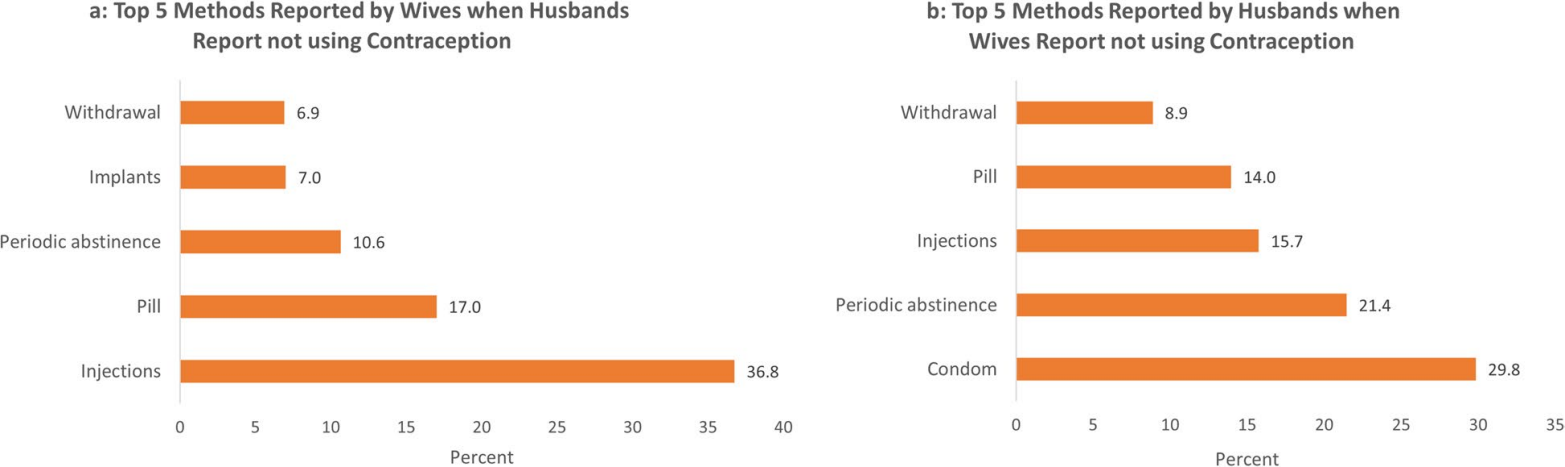
**a** Top 5 methods reported by wives when husbands report not using contraception. b Top 5 methods reported by husbands when wives report not using contraception

To understand the extent of discordance we compare the reported contraceptive method of men and women in monogamous relationships from the DHS couples file. Discordance is defined as any instance where the response from the women did not match that of her husband i.e. both partners report using a method but name different methods or one partner reports using and the other does not (Figs 6, 7 and 8b).

We find that for matched partners there is a high level of discordance in reported contraceptive method with more than a fifth of couples with conflicting reports in all regions. When narrowing in on the source of the discordance, the majority was due to one partner (more often the husband) reporting usage while the other did not. We then specifically focus on the methods that are mentioned in the instances where one partner reports not using contraception. For wives, the leading choice was injectables while for husbands it was the condom. The former mismatch is a likely indicator of clandestine usage by women because there is no physical evidence after using injectables and the latter likely indicative of extra marital relationships where husbands use condoms with other women but not their wives.

### Determinants of barrier method usage

Table 2 presents multilevel logistic regressions predicting the likelihood of using barrier methods, by gender. National HIV prevalence at the time of the survey is correlated with a greater likelihood of using barrier methods for women but the coefficients are not statistically significant. For men, there are significantly lower odds of condom use in countries with high HIV prevalence. There are no statistically significant differences in condom usage by level of AIDS knowledge for both males and females.

**Table 2:** Multilevel logistic regression parameter estimates of barrier method usage (standard errors given in brackets)

Parameter	**Male**			**Female**		
	**Model 0**	**Model I**	**Model II**	**Model 0**	**Model I**	**Model II**
**Fixed effects**						
**Constant**	1.505* (0.185)	2.372*** (0.219)	50.308*** (0.530)	0.208*** (0.231)	0.130*** (0.500)	1.161 (0.444)
**HIV**						
Low		1	1		1	1
High		0.420*** (0.248)	0.248*** (0.209)		1.567 (0.414)	1.174 (0.147)
**AIDS Knowledge**						
*Low (ref)*						
Medium			0.956 (0.048)			0.885 (0.085)
High			0.961 (0.094)			1.068 (0.068)
Survey Year						
*1990* - *1994 (ref)*		1	1		1	1
1995 - 1999		0.922 (0.087)	1.111 (0.155)		0.653 (0.513)	0.553 (0.459)
2000 - 2004		0.978 (0.099)	1.124 (0.165)		1.799 (0.348)	1.602* (0.210)
2005 - 2009		1.090 (0.110)	1.305 (0.191)		2.003 (0.384)	1.715* (0.243)
2010 - 2014		0.838* (0.084)	0.866 (0.140)		1.510 (0.368)	1.199 (0.218)
2015 - 2019		0.932 (0.084)	1.136 (0.222)		1.125 (0.436)	1.091 (0.283)
**Marital Status**						
*Never married (ref)*			1			1
In union			0.105*** (0.338)			0.359*** (0.080)
Formerly married			0.791 (0.298)			0.718*** (0.049)
**Education**						
*No education (ref)*			1			1
Primary			1.120 (0.129)			1.426*** (0.107)
Secondary			1.118 (0.161)			1.868*** (0.114)
Higher			1.024 (0.198)			2.320*** (0.081)
**Age Group**						
*15-19 (ref)*			1			1
20-24			0.609*** (0.085)			0.804* (0.086)
25-29			0.505*** (0.119)			0.852 (0.157)
30-34			0.422*** (0.156)			0.861 (0.227)
35-39			0.429*** (0.172)			1.056 (0.283)
40-44			0.406*** (0.172)			0.963 (0.295)
45-49			0.380*** (0.181)			1.172 (0.432)
50-54			0.433*** (0.211)			-
55-59			0.320*** (0.259)			-
**Wealth Status**						
*Poorest (ref)*			1			1
Poorer			0.934 (0.090)			0.998 (0.076)
Middle			0.936 (0.158)			0.970 (0.064)
Richer			0.877 (0.163)			0.968 (0.094)
Richest			0.964 (0.144)			1.152 (0.135)
**Religion**						
*No religion (ref)*			1			1
Christian			0.958 (0.099)			1.001 (0.110)
Muslim			0.903 (0.162)			0.741* (0.135)
Traditionalist/Animist			1.102 (0.216)			1.221 (0.221)
Other			1.218 (0.128)			1.528* (0.200)
**Type of Place of Residence**						
*Urban (ref)*			1			1
Rural			0.823* (0.090)			0.869** (0.052)
**Number of Living Children**						
*None (ref)*			1			1
1–3 children			0.442*** (0.151)			0.209*** (0.158)
4–6 children			0.316*** (0.142)			0.100*** (0.136)
7 or more			0.239*** (0.148)			0.049*** (0.173)
**Random Effects**						
Country	0.854*** (0.247)	0.999*** (0.288)	1.215*** (0.351)	1.707*** (0.434)	1.685*** (0.428)	1.412*** (0.359)
ICC - Country	0.206	0.233	0.270	0.342	0.339	0.300
AIC	497,049,330	493,814,457	600,107,170	542,768,349	537,759,941	575,958,395
BIC	497,049,347	493,814,473	600,107,187	542,768,366	537,759,957	575,958,411
Log Likelihood	497,049,328	493,814,455	600,107,168	542,768,347	537,759,939	575,958,393
Deviance	-994098656.4	-987628909.8	-1,200214336	-1085536695	-1075519877	-1151916785

*ICC* Intra-level correlation coefficient, *AIC* Akaike information criterion, *BIC* Bayesian information criterion

*** *p* < 0.001 ** *p* < 0.01 * *p* < 0.05

As observed in the analysis of time trends, both women and men in union are significantly less likely to be using barrier methods that those not in union.

The regression indicates that usage of barrier methods increased significantly for women in the 2000s relative to the early 1990s for women. For men there was an increase in the odds of condom use during the period but the increase was not statistically significant. For women the odds of condom use in the 2010s remained higher than the early 1990s, but the odds were not significant. For men, there are decreased odds in the early 2010s and higher odds in the late 2010s but the difference was not statistically significant.

## Discussion

This paper conducts a descriptive analysis of trends in contraceptive use and method mix dynamics in sub-Saharan Africa, with a focus on whether preferences for condom usage have changed over time. While there may be different motivations for the use of contraceptives, the following two stand out; i.e., preventing a pregnancy, or preventing sexually transmitted infections (STIs). However, method preference may be influenced by different motivations, including beliefs and knowledge of efficacy of the method, conceptions and or misconceptions, and the disease environment especially with respect to STIs. For example, there may be high preference for the use of barrier methods specifically condoms in settings where there is high prevalence of STIs.

The descriptive analyses indicate that contraceptive method mix has changed over time and the pattern differs by gender. Generally, the use of the pill has declined over time as has traditional methods such as periodic abstinence while the use of condoms, injections and implants have increased. The multivariate analysis in this paper focuses on condom usage only and thus subsequent research can explore in more detail the time trends in the usage of other methods.

The increase in condom use in the 2000s may be attributed to greater demand for STI prevention in addition to preventing unwanted pregnancies. Condom use, after that increase, has declined in more recent years which is consistent with findings from other surveys indicating that condom usage is on the decline [13, 14]. One possible explanation for the decline in condom use in recent times could result from optimism arising out of the immunosuppressive effect of antiretrovirals thus creating a false impression of control of HIV/AIDS.

However, if the time trends observed in condom usage was solely driven by the HIV pandemic, then controlling for national HIV prevalence in the model would have explained away the rise and fall pattern observed in the graphs. The persistent significant coefficients recorded for the 2000s despite the controls indicate additional research is needed to understand the drivers of these time trends. The negative odds of using condoms in high prevalence countries for males, which can be indicative of a reverse causality relationship where prevalence is high because males are less likely to use condoms, also warrants further study.

The increased use of long-acting methods such as implants and injections particularly in the more recent periods may be due to the increased active promotion of such methods as more effective methods of contraception than the pill which has seen consistent decline over time. While reasons for the significant decline in the use of the pill have not been investigated in this paper, evidence from other studies suggest that women are moving away from the use of the pill because of reported side effects [15, 16]. The IUD has also been reported to have serious side effects and this might also account for its declining use [17, 18].

The time trend analyses highlight the importance of expanding the focus of contraceptive use studies beyond women in this context as we see differing trends for men.

Men are more likely to report condom use than women while women are more likely to report injection usage. The discordance in terms of the differences in the reported condom usage between males and females is also interesting and may possibly be due to some males using condoms with partners outside of their spouses or regular partners. The increasing usage of injections for females in this context are suggestive of a demand for contraceptive methods that women can discreetly use without the involvement of their partners. These finding highlight the need to continue to increase women’s access to all the available contraceptive methods, so they choose the method that best suits their needs.

## Conclusions

This paper uses Demographic and Health Survey (DHS) data from 32 countries to conduct extensive analysis of trends in the use of barrier versus non-barrier contraceptive use in sub-Saharan Africa. The paper examines how contraceptive method mix dynamics have changed over time and whether the trends differ by marital status and gender using cross-tabulations. It further examines the determinants of method choice using logistic regressions focusing on HIV prevalence and HIV knowledge as explanatory factors.

The findings indicate that the use of barrier methods, most markedly for unmarried women and men, rose significantly between the late 1980s and late 2000s in the region in tandem with trends in HIV prevalence. The results further show marked differences in method mix by gender with men being more likely to report barrier method use than women. The World Health Organisation has cautioned that the world faces increasing HIV prevalence due to service disruptions after COVID-19 as such declining use of barrier methods will have implications in this new post-pandemic era.

## Data Availability

The datasets analysed during the current study are available from https://dhsprogram.com/data and http://aidsinfo.unaids.org.

## Appendix

**Table 3:** List of surveys

**No.**	**Country**	**Year of survey**									
		**Female**					**Male**				
1	Benin	1996	2001	2006	2012	2017	1996	2001		2012	2017
2	Burkina Faso	1999	2003	2010			1999	2003	2010		
3	Burundi	2010	2017				2010	2017			
4	Cameroon	1998	2004	2011			1998		2011		
5	Chad	1997	2004	2015			1997	2004	2015		
6	Comoros	1996	2012				1996	2012			
7	Congo	2005	2011								
8	Congo DR	2007	2013					2013			
9	Cote D'Ivoire	1994	1999	2012			1994	1999	2012		
10	Ethiopia	2000	2005	2011	2016		2000	2005	2011	2016	
11	Gabon	2000	2012				2000	2012			
12	Ghana	1998	2003	2008	2014		1998	2003	2008	2014	
13	Guinea	1999	2005	2012	2018		1999		2012	2018	
14	Kenya	1998	2003	2009	2014		1998	2003	2009	2014	
15	Lesotho	2004	2009	2014				2009	2014		
16	Liberia	2007	2013				2007	2013			
17	Madagascar	1997	2004	2009				2004	2009		
18	Malawi	1999	2004	2010	2016		1999	2004	2010	2016	
19	Mali	1995	2001	2006	2012	2018	1995	2001	2006	2012	2018
20	Mozambique	1997	2003	2011			1997		2011		
21	Namibia	2000	2006	2013			2000	2006	2013		
22	Niger	1998	2006	2012			1998	2006	2012		
23	Nigeria	2003	2008	2013	2018			2008	2013	2018	
24	Rwanda	2000	2005	2010	2015		2000		2010	2015	
25	Senegal	1997	2005	2010	2016			2005	2010	2016	
26	Sierra Leone	2008	2013				2008	2013			
27	South Africa	1998	2016					2016			
28	Tanzania	1996	2000	2005	2010		1996		2005	2010	
29	Togo	1998	2013				1998	2013			
30	Uganda	1995	2001	2006	2011	2016		2001	2006	2011	2016
31	Zambia	1996	2002	2007	2014	2018	1996	2002	2007	2014	2018
32	Zimbabwe	1994	1999	2006	2011	2015	1994	1999	2006	2011	2015

## Footnotes

1 Missing cases were not excluded at the univariate level but are excluded for the multivariate analysis.

2 The detailed methodology is explained here: (https://dhsprogram.com/pubs/pdf/DHSM4/DHS6_Sampling_Manual_Sept2012_DHSM4.pdf). Estimated population at the period of survey were derived from the World Population Prospects (https://population.un.org/wpp/; assessed on 13th May 2023). Data on total number of women interviewed were also derived from the respective published survey reports. The same denormalization approach was adopted and done separately for the male sample of 15–59 years.
